# Structure of the GDP-bound state of the SRP GTPase FlhF

**DOI:** 10.1107/S2053230X24000979

**Published:** 2024-02-20

**Authors:** Anita Dornes, Christopher-Nils Mais, Gert Bange

**Affiliations:** a Center for Synthetic Microbiology (SYNMIKRO) and Department of Chemistry, University of Marburg, Karl-von-Frisch-Strasse 14, 35043 Marburg, Germany; b Molecular Physiology of Microbes, Max Planck Institute for Terrestrial Microbiology, Karl-von-Frisch-Strasse 14, 35043 Marburg, Germany; Weizmann Institute of Science, Israel

**Keywords:** flagellar assembly, SRP GTPases, GTPases, FlhF, nucleotides

## Abstract

The GTPase FlhF, a signal recognition particle (SRP)-type enzyme, is pivotal for spatial–numerical control and bacterial flagella assembly across diverse species, including pathogens. This study presents the X-ray structure of FlhF in its GDP-bound state at a resolution of 2.28 Å. The structure exhibits the classical N- and G-domain fold, consistent with related SRP GTPases such as Ffh and FtsY. Comparative analysis with GTP-loaded FlhF elucidates the conformational changes associated with GTP hydrolysis. These topological reconfigurations are similarly evident in Ffh and FtsY, and play a pivotal role in regulating the functions of these hydrolases.

## Abstract 2

This study presents the X-ray structure of FlhF in its GDP-bound state at a resolution of 2.28 Å, exhibiting the classical N- and G-domain fold. Comparative analysis with GTP-loaded FlhF elucidates the conformational changes associated with GTP hydrolysis.

## 1. Introduction

Flagella are bacterial organelles of locomotion that enable bacteria to move across chemical gradients (Chevance & Hughes, 2008 ▸). Bacteria exhibit variations in both the quantity and the positioning of their flagella, which manifest in consistent arrangements on the cell surface, known as flagellation patterns (reviewed in Schuhmacher *et al.*, 2015 ▸; Kazmierczak & Hendrixson, 2013 ▸). Despite the vast diversity among bacterial species, only a limited number of these patterns are observed. An accurate flagellation pattern is essential to enable motility and is also intricately connected to processes such as biofilm formation and the pathogenicity of bacteria that contribute to diseases and possess flagella.

**Figure 1:**
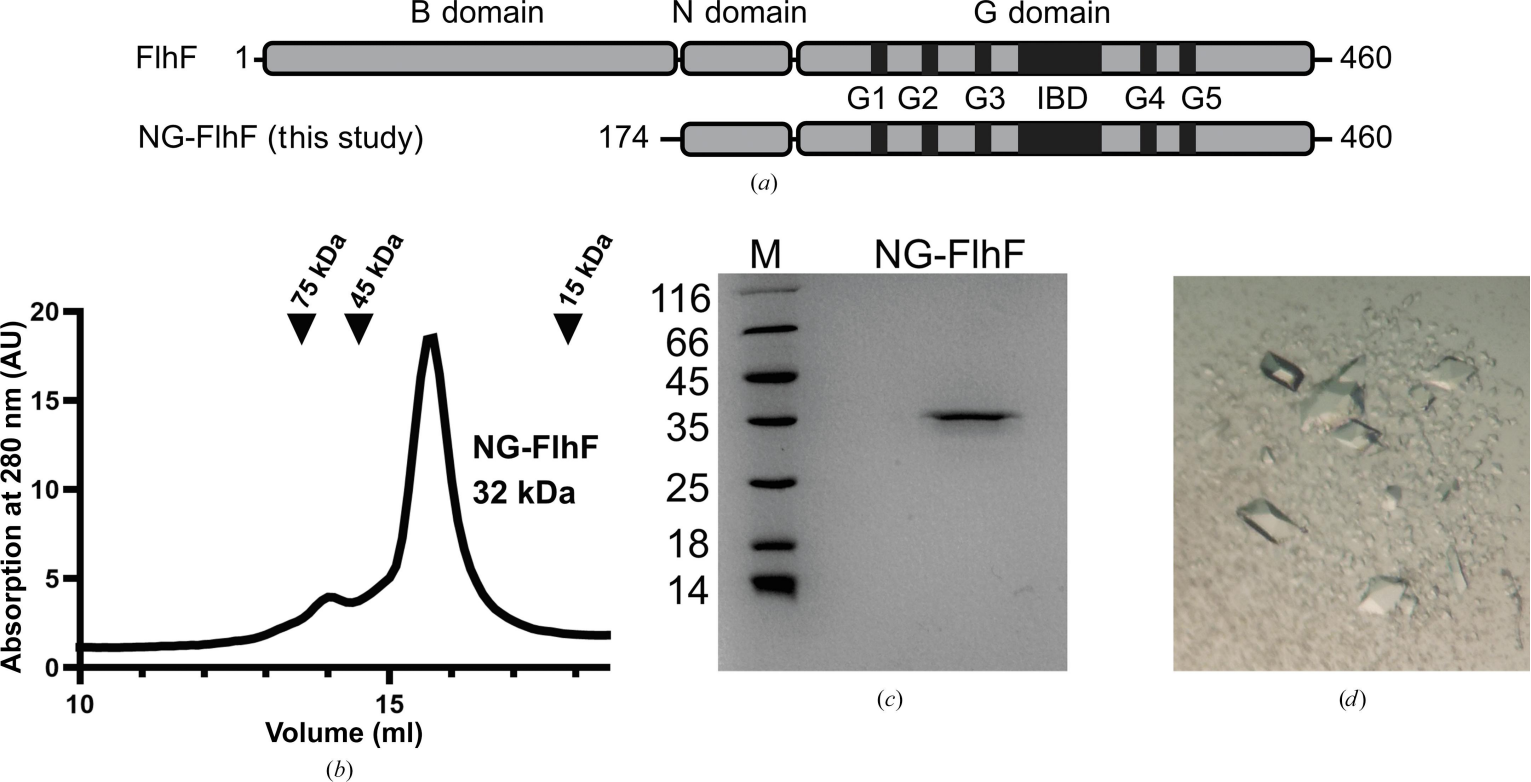
(*a*) Domain architecture of FlhF and the construct used in this study. (*b*) Analytical size-exclusion chromatography reveals the monomeric state of FlhF in the presence of GDP. (*c*) SDS–PAGE of NG-FlhF. (*d*) Bipyramidal crystals of NG-FlhF.

The GTPase FlhF is central to the correct spatial positioning of flagella within different bacterial species (summarized in Schuhmacher *et al.*, 2015 ▸). FlhF, together with SRP54 (or Ffh) and FtsY, belongs to an evolutionarily conserved protein family with only three members: the signal recognition particle (SRP) GTPases (Leipe *et al.*, 2002 ▸, 2003 ▸). The three SRP GTPases share a conserved NG domain, with the N domain comprising an α-helical bundle and the G domain being a GTPase with an α–β–α insertion termed the I-box (Bange, Petzold, Wild, Parlitz *et al.*, 2007 ▸; Montoya *et al.*, 1997 ▸; Freymann *et al.*, 1997 ▸; Fig. 1 ▸ *a*). Within the G domain, five conserved G elements ensure the proper functioning of the GTPase. The G1 element [G*xxx*GGK(S/T), where *x* stands for any amino acid], also known as the P-loop, forms the oxyanion hole for the α- and β-phosphates of the nucleotide (reviewed by Saraste *et al.*, 1990 ▸). In SRP GTPases, the G2 element contains a DT*x*R motif, with threonine coordinating the γ-phosphate and the catalytic magnesium ion. This element also features an arginine finger that facilitates GTP hydrolysis (Focia *et al.*, 2004 ▸; Bange *et al.*, 2011 ▸; Egea *et al.*, 2004 ▸). G3 (D*xx*G) coordinates the magnesium ion and the nucleophilic water. Nucleotide recognition is facilitated by the G4 element [(N/T)(K/Q)*x*D], which utilizes its aspartate to sense N1 and N2 of guanosine. Finally, the topologically conserved G5 element [G*x*(G/S)(K/E/Q) in the SRP GTPases] coordinates the base and ribose, enclosing the nucleotide-binding site (Leipe *et al.*, 2002 ▸).

SRP54 and FtsY are essential components of the universally conserved SRP system that facilitates the co-translational transport of secretory and membrane proteins to the plasma membrane and endoplasmic reticulum in prokaryotes and eukaryotes, respectively (reviewed, for example, by Grudnik *et al.*, 2009 ▸; Saraogi & Shan, 2014 ▸; Njenga *et al.*, 2023 ▸). The SRP, a ribonucleoprotein particle, comprises a core formed by the SRP54 protein and its binding site on SRP RNA that recognizes signal sequences in emerging proteins. Interaction with the receptor FtsY at the membrane facilitates the transfer of the ribosome nascent-chain complex (RNC) to the translocation channel. This process is coordinated by a complex of SRP and its receptor, which is formed through a GTP-dependent interaction of the NG domains of SRP54 and FtsY in a nearly symmetric heterodimer. Within this NG-domain complex, both GTPases mutually stimulate each other’s activity, which leads to dissociation of the complex and enables the next round of SRP-dependent protein targeting.

In contrast, FlhF forms a GTP-dependent homodimer via its NG domains with a high degree of structural similarity to the heterodimer formed by the NG domains of SRP54 and FtsY (Bange, Petzold, Wild, Parlitz *et al.*, 2007 ▸; Bange, Petzold, Wild & Sinning, 2007 ▸). The GTPase activity of the FlhF homodimer and its dissociation into a GDP-bound monomer is stimulated by the protein FlhG, which serves as numerical regulator of flagella biosynthesis (Bange *et al.*, 2011 ▸; Kusumoto *et al.*, 2008 ▸; Rossmann *et al.*, 2015 ▸). However, a structural description of a GDP-bound monomer of the GTPase FlhF is still elusive. In this report, we present the biochemical and structural analysis of a GDP-bound monomer of FlhF from the polar-flagellated bacterium *Shewanella putrefaciens* CN32.

## 2. Materials and methods

### 2.1. Protein production

Our construct was engineered using multiple sequence alignments and structure-prediction-based decision-making and encompassed amino acids 174–460 (NG-FlhF) of *S. putrefaciens* CN32 FlhF (UniProt A4Y8J9; Supplementary Fig. S1). The corresponding gene (*Sputcn32_2561*) was subsequently amplified by polymerase chain reaction using the Expand High Fidelity PCR system (Roche) and inserted into a pET-24d vector (Novagen) via NcoI/XhoI restriction sites (Supplementary Fig. S1). The construct, which was validated by sequencing, features a hexahistidine (His_6_) tag at the C-terminus of the protein. *Escherichia coli* strain BL21 (DE3) (Novagen) was employed for NG-FlhF expression; cells cultured in lysogeny broth (LB) medium were supplemented with 1.5%(*w*/*v*) d-(+)-lactose monohydrate for 16 h at 303 K.

### 2.2. Protein purification

The expression cultures were harvested by centrifugation (6000*g* for 15 min at 293 K) and the pellets were resuspended in 10 ml lysis buffer per gram of cells and processed using an M-110L Microfluidizer (Microfluidics). The lysis buffer consisted of 20 m*M* Tris pH 8.5, 500 m*M* NaCl, 10 m*M* MgCl_2_, 10 m*M* KCl, 10%(*v*/*v*) glycerol. The lysate underwent clarification by centrifugation (125 000*g* for 30 min at 293 K) using a Ti-45 rotor (Beckmann) and was applied onto a 1 ml HisTrap HP column (GE Healthcare). The column underwent an initial wash with five column volumes of lysis buffer containing 40 m*M* imidazole pH 8.5. Protein elution took place in lysis buffer containing 500 m*M* imidazole pH 8.5. Subsequently, the protein was concentrated to approximately 30 mg ml^−1^ using an Amicon Ultracel-10K (Millipore) and was subjected to size-exclusion chromatography using an Superdex 75 XK 26/600 column (GE Healthcare) in the same buffer as above but without imidazole. Protein-containing fractions were combined and concentrated to 10 mg ml^−1^.

### 2.3. Crystallization, data collection and structure determination

Crystallization was performed by the sitting-drop method at 20°C in 250 nl drops consisting of equal parts of 1.8 m*M* protein solution pre-incubated with 5 m*M* GDP and precipitation solution. The crystallization condition was 1 *M* lithium chloride, 0.1 *M* citric acid pH 5.0, 20% PEG 6000. Data were collected under cryogenic conditions on beamline ID23-1 at the European Synchrotron Radiation Facility (ESRF), Grenoble, France. The data were processed with *XDS* and were scaled with *XSCALE* (Kabsch, 2010 ▸). The structure was determined by molecular replacement with *Phaser* (McCoy *et al.*, 2007 ▸) utilizing the structure of GTP-bound *Bacillus subtilis* FlhF (PDB entry 2px3; Bange, Petzold, Wild, Parlitz *et al.*, 2007 ▸). It was then manually built in *Coot* (McCoy *et al.*, 2007 ▸) and refined with *Phenix* 1.18.2 (Liebschner *et al.*, 2019 ▸). GDP was only added in the final refinement step, assuring its fit into the unbiased electron density.

## 3. Results and discussion

FlhF consists of an N-terminal B domain, which is widely disordered outside its cellular context, followed by the NG domain (Fig. 1 ▸ *a*). The NG domain of FlhF, denoted NG-FlhF, was anticipated to span amino acids 174–460 (Fig. 1 ▸ *a*) from predictions based on multiple sequence alignments and secondary-structure analyses (not shown). Expression of NG-FlhF yielded approximately 40 mg per litre of culture (equivalent to 8 g of cells) and protein purification followed standard protocols described previously (Bange, Petzold, Wild & Sinning, 2007 ▸). Analytical size-exclusion chromatography (SEC) revealed the presence of a monomer (Fig. 1 ▸ *b*). The purity of the protein before crystallization exceeded 95%, as determined by Coomassie-stained SDS–PAGE (Fig. 1 ▸ *c*).

Initial crystals of NG-FlhF were grown using sitting drops, where the reservoir solution consisted of 0.1 *M* citric acid pH 4.0, 1.6 *M* ammonium sulfate with a final pH of 5.0 (Core 2, F12; NeXtal Classics Suite). Bipyramidal crystals appeared within two days (Fig. 1 ▸ *d*). Diffraction data were collected at 2.28 Å resolution on beamline ID23-1 at the ESRF in Grenoble. The crystals belonged to space group *P*6_1_22 and contained one molecule of NG-FlhF per asymmetric unit. The structure was determined by molecular replacement using the structure of NG-FlhF from *B. subtilis* (Bange, Petzold, Wild, Parlitz *et al.*, 2007 ▸), manually built in *Coot* and refined to *R*_work_ and *R*_free_ values of 0.24 and 0.27, respectively (Table 1 ▸).

**Figure 2:**
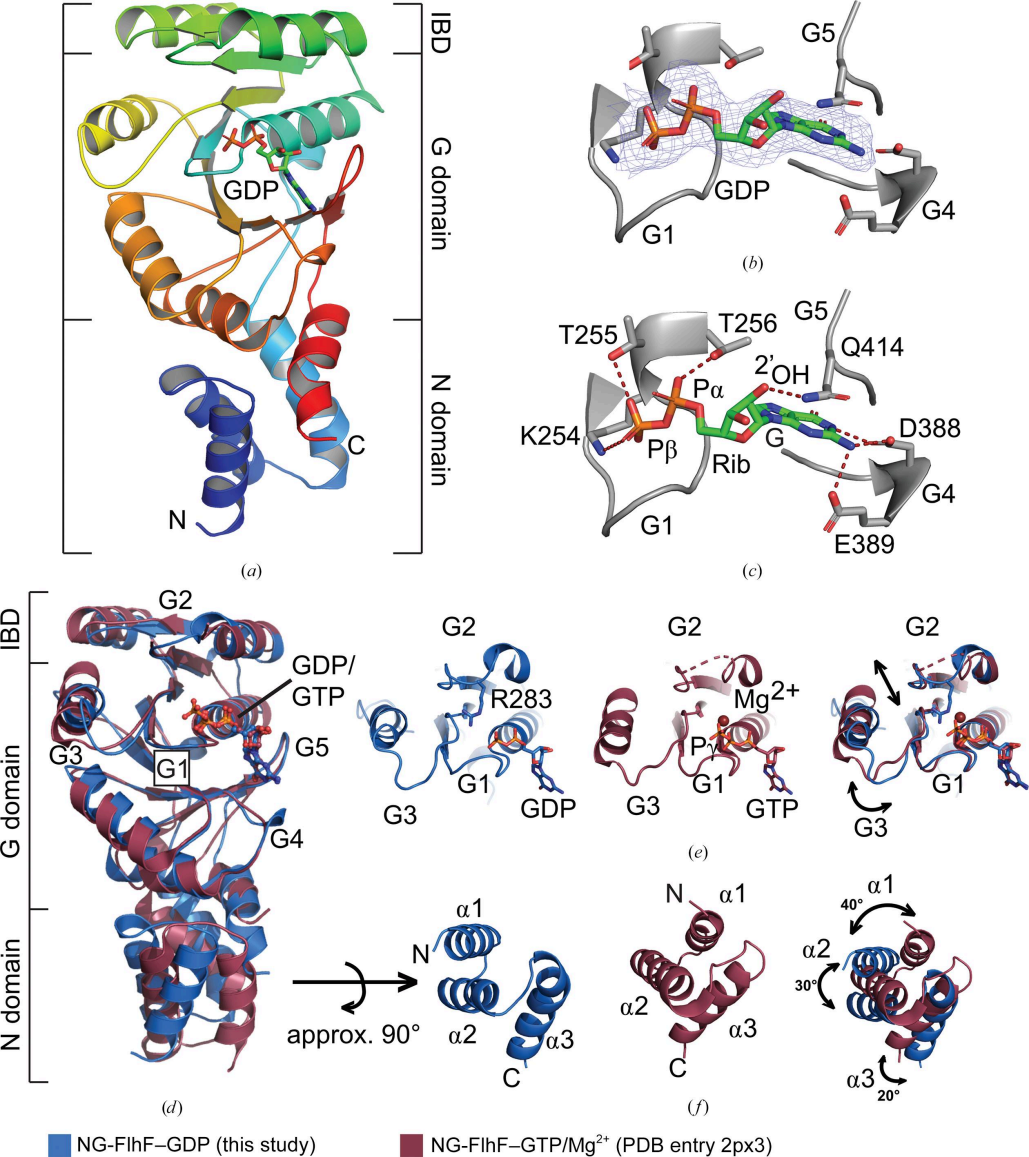
(*a*) Crystal structure of NG-FlhF. (*b*) Electron density reveals bound GDP. (*c*) A close-up view of the active site reveals the canonical GTPase motifs. (*d*) Superposition of our GDP-bound NG-FlhF (blue) with GTP/Mg^2+^-bound NG-FlhF (red). (*e*) A side-by-side view of the G2 and G3 loops reveals a tightening of the active site. (*f*) The N domain rotates during the transition between GDP-bound and GTP-bound states.

Amino-acid residues 183–437 of NG-FlhF could be unambiguously assigned into the electron-density map and represent almost all of the residues of the protein construct employed in this study. The structure of NG-FlhF reveals that of a classical NG domain of the SRP-GTPase family (Fig. 2 ▸ *a*). The N domain features a three-helical bundle and the G domain exhibits the Ras-GTPase fold with an α–β–α insertion, known as the insertion box domain (IBD) (Fig. 2 ▸ *a*).

Density which could unambiguously be assigned to GDP was found in the active center of NG-FlhF (Fig. 2 ▸ *b*). The GDP is coordinated by residues of the G1, G4 and G5 elements, which are conserved among the SRP GTPase family (Bange, Petzold, Wild, Parlitz *et al.*, 2007 ▸). In the GDP-bound state of FlhF, the guanine base of the nucleotide is coordinated by Asp388 and Glu389 of the G4 element (Fig. 2 ▸ *c*). Gln414 of the G5 element hydrogen-bonds to the 2′-OH group of the ribose moiety. The α- and β-phosphates of GDP are coordinated by Lys254, Thr255 and Thr256 of the G1 element. Density for a magnesium ion was not present within the active site of NG-FlhF. Overall, our study presents the first structure of an NG domain of FlhF in the GDP-bound state.

In the next step, we wanted to analyze the conformational differences between the GDP-bound and GTP-bound states of FlhF. Thus, we superimposed the monomeric GDP-bound state of NG-FlhF (this study) with its GTP-bound state, in which the protein exists as a homodimer (Bange *et al.*, 2011 ▸; Bange, Petzold, Wild, Parlitz *et al.*, 2007 ▸). In general, the structures superimpose well, with a root-mean-square deviation (r.m.s.d.) of 1.332 Å over 1085 atoms (Fig. 2 ▸ *d*). However, a visual inspection revealed substantial differences, which primarily localize within the active site. Most prominently, Arg283 within the G2 loop is properly ordered in our structure, coordinating the β-phosphate of the GDP (Supplementary Fig. S2). In the GTP-Mg-bound state, this residue could not be resolved, hinting at a high degree of flexibility. Using this sensing mechanism, the G2 loop and the adjacent α-helix are pulled 4 Å towards the nucleotide (Fig. 2 ▸ *e*). As a second prominent change, the G3 loop rotates to a position 2.5 Å closer to the active site. This movement indirectly induces conformational changes in the N domain, as discussed in the next section.

A difference in the spatial orientation of the helical bundle forming the N domain can be noted. The binding of GDP introduces a rotation of 20–40° of the helices of the bundle in relation to its center. Due to this unequal twist, the mutual spatial arrangement of the helices is also marginally distorted (Fig. 2 ▸ *f*). As a conclusion, the GDP-bound and GTP-bound states of FlhF can be distinguished by the topology of their N domains. As this domain has been shown to affect correct flagellar assembly, an as yet unexplored signaling mechanism can be hypothesized (Li *et al.*, 2022 ▸).

Our structure of FlhF could be superimposed onto structures of the other two members of the SRP GTPases, Ffh and FtsY, from *Thermus aquaticus* with r.m.s.d. values ranging from 1.920 to 3.186 Å (Table 2 ▸; Gawronski-Salerno *et al.*, 2007 ▸; Freymann *et al.*, 1999 ▸; Gawronski-Salerno & Freymann, 2007 ▸; Bange, Petzold, Wild, Parlitz *et al.*, 2007 ▸).

**Figure 3:**
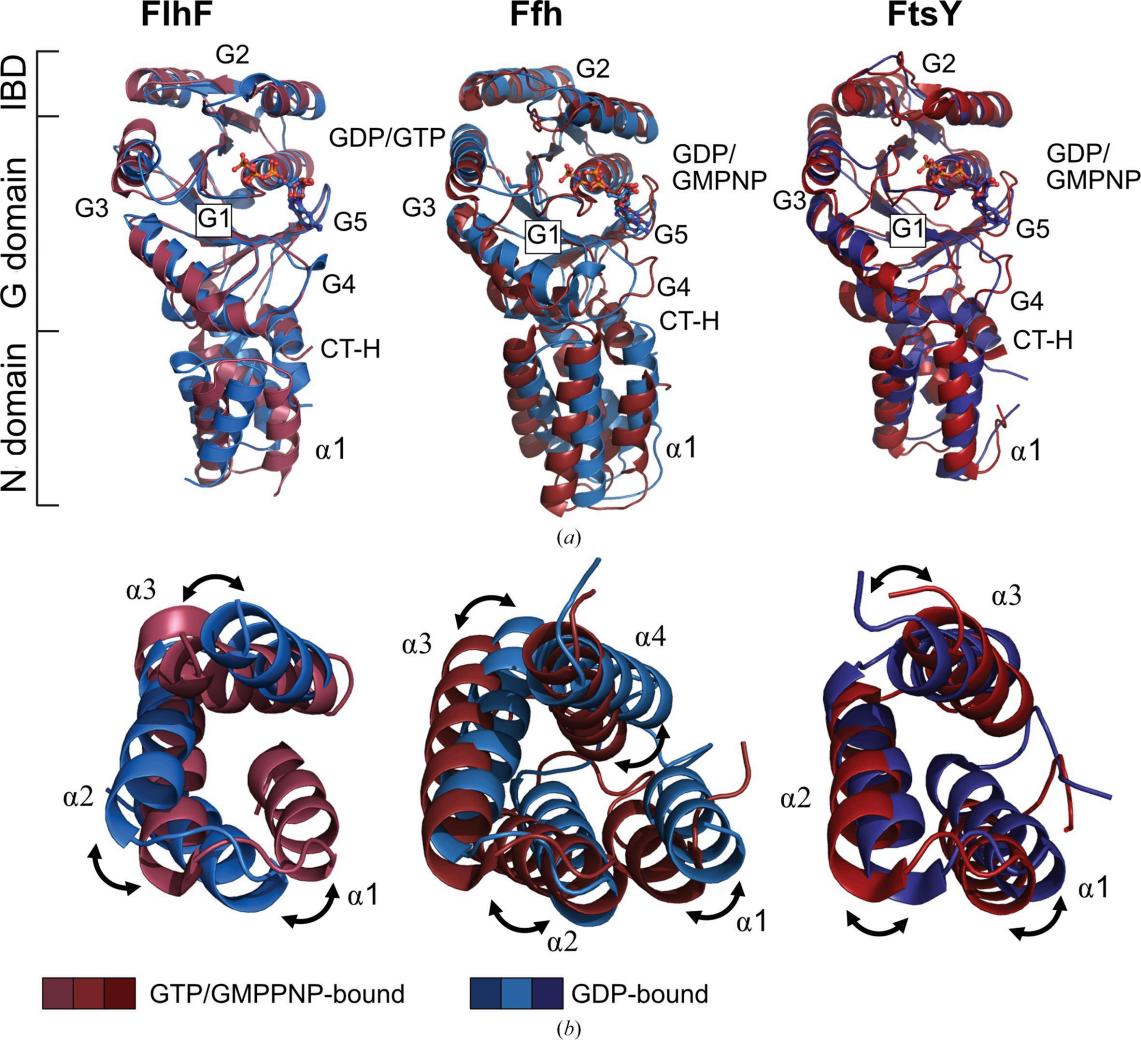
(*a*) Crystal structures of GDP-bound (blue) and GTP-bound or GMPPNP-bound (red) states of FlhF, Ffh and FtsY. Comparison reveals conserved rearrangements in the G2 and G3 elements, as well as larger modifications in the G4 and G5 regions within the SRP GTPases. (*b*) Close-up of the superimposition of the N-domain helical bundle. Topological changes depending on the nucleotide-binding state are conserved within this family of GTPases.

Utilizing the nonhydrolyzable GTP analog GMPPNP and GDP, both states of these GTPases have been characterized, enabling a comparison with the findings of our study (Fig. 3 ▸ *a*). In the case of FlhF, G1 remains relatively stable. Additionally, the adjustments observed in the G2 and G3 elements are mirrored in the SRP GTPases. However, the G4 and G5 elements undergo significant rearrangements in both SRP GTPases when transitioning between the GDP-loaded and GMPPNP-loaded states. Given the crucial role of these loops in formation of the homoheterodimeric complex, differentially bound nucleotides are expected to exert a stronger effect, possibly reflecting an evolutionary adaptation to more robustly prevent SRP complex formation compared with erroneous FlhF complex formation. A comparison of the N-terminal domain reveals that the topological shifts in FlhF are conserved in Ffh and FtsY (Fig. 3 ▸ *b*). Despite variations in helical composition and arrangement in the N domain, angular twists in individual helices deform the helical bundle, resulting in an altered overall appearance. Notably, FlhF angular twists of α2 move in the opposite direction with regard to Ffh and FtsY, potentially resulting from differing targets of the signal.

The C-terminal helix (CT-H) localizes within the interface of the N and G domains in all three SRP GTPases, primarily engaging in hydrophobic interactions. Comparative analysis across distinct states reveals a synchronized movement of the C-terminal helix and the N domain (Fig. 3 ▸ *a*). Therefore, the CT-H could be viewed as an additional helix of the N domain of SRP GTPases. It is noteworthy that the N domains of the SRP GTPases Ffh and FtsY comprise four helices in the GDP-bound state, with helix α-N1 being expelled from each helical bundle upon Ffh–FtsY complex formation (Egea *et al.*, 2004 ▸; Focia *et al.*, 2004 ▸; Wild *et al.*, 2016 ▸). In contrast, the N domain of FlhF consistently exhibits three α-helices alongside the C-terminal domain independent of its nucleotide-dependent oligomeric state (Bange, Petzold, Wild, Parlitz *et al.*, 2007 ▸; this study). The potential biological significance of this discrepancy awaits further investigation. Overall, our structure reveals a previously undescribed state of the GTPase cycle of FlhF, offering insight into topological rearrangements of the active site and N domain.

## Supplementary Material

PDB reference: GDP-bound state of *Shewanella putrefaciens* FlhF, 8r9r

Supplementary Figures. DOI: 10.1107/S2053230X24000979/nd5006sup1.pdf

## Figures and Tables

**Table 1:** Data-collection and refinement statistics Values in parentheses are for the highest resolution shell. Data were collected on beamline ID23-1 at the ESRF.

Data collection	
Space group	*P*6_1_22
*a*, *b*, *c* (Å)	73.92, 73.92, 159.96
α, β, γ (°)	90, 90, 120
Wavelength (Å)	0.885600
Resolution (Å)	40.97–2.42 (2.507–2.420)
*R*_merge_	0.1142 (2.228)
〈*I*/σ(*I*)〉	21.75 (1.49)
Completeness (%)	99.84 (99.01)
Multiplicity	35.8 (37.1)
CC_1/2_	1 (0.754)
Refinement	
Resolution (Å)	40.97–2.42 (2.507–2.420)
No. of reflections	10470 (1001)
*R*_work_/*R*_free_	0.24/0.27
No. of atoms	
Total	1966
Protein	1936
Ligand/ion	29
Water	1
*B* factors (Å^2^)	
Overall	55.76
Protein	55.67
Ligand/ion	61.14
Water	69.31
R.m.s. deviations	
Bond lengths (Å)	0.009
Bond angles (°)	1.26
Ramachandran statistics	
Favored (%)	96.05
Allowed (%)	3.16
Outliers (%)	0.79

**Table 2:** Superimposition of FlhF, Ffh and FtsY

	FlhF	Ffh	FtsY
GDP-bound			
PDB code	8r9r †	2ng1	2iyl
R.m.s.d. (Å)	N/A	2.523	3.186
Organism	*Shewanella putrefaciens*	*Thermus aquaticus*	*Thermus aquaticus*
GTP/GMPPNP-bound			
PDB code	2px3	2j7p	2j7p
R.m.s.d. (Å)	1.355	1.920	2.566
Organism	*Bacillus subtilis*	*Thermus aquaticus*	*Thermus aquaticus*

† This study.
