# Supplementary material for: Structure of the GDP-bound state of the SRP GTPase FlhF
Source: Acta Crystallogr F Struct Biol Commun. 2024 Feb 20;80(Pt 3):53–8. doi: 10.1107/S2053230X24000979 (PMC10910532; doi:10.1107/S2053230X24000979)
Supplement: Supplementary file 1 [file f-80-00053-sup1.pdf]

STRUCTURAL BIOLOGY COMMUNICATIONS

Volume 80 (2024)

Supporting information for article:

Structure of the GDP-bound state of the SRP GTPase FlhF

Anita Dornes, Christopher-Nils Mais and Gert Bange

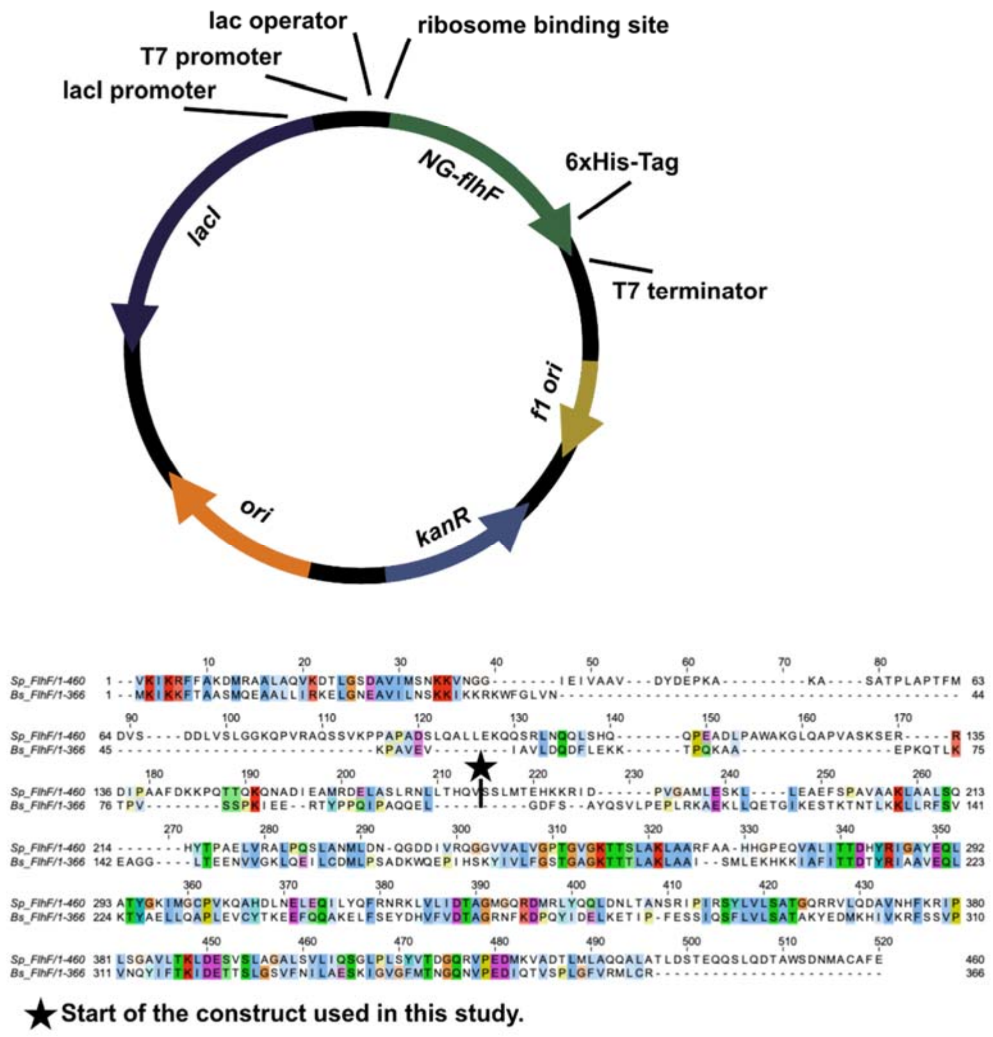

Figure S1 Plasmid map employed for this study. **B.** Sequence alignment of *S. putrefaciens* and *Bacillus subtilis* FlhF.

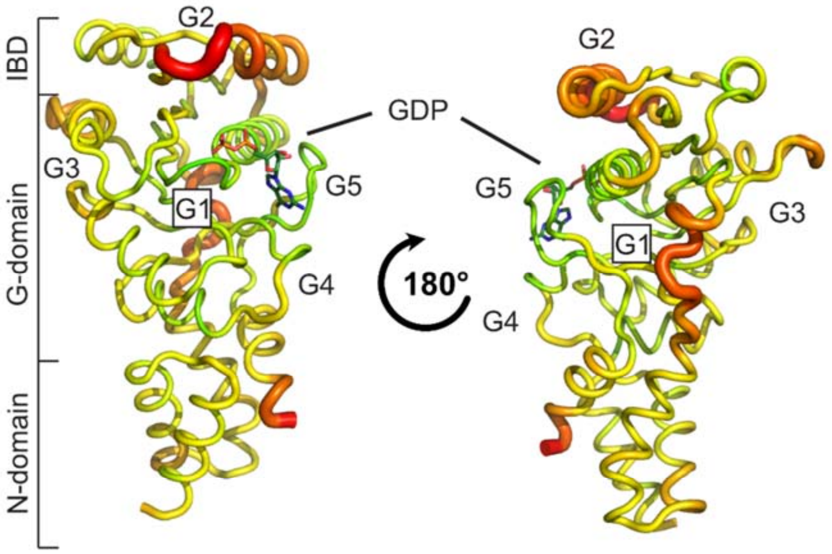

Figure S2 B-factors of the GDP bound NG-FlhF published with this study. B-factors range from 44 (yellow, thin) to 91 (red, thick).
